# Heat stroke with bimodal rhabdomyolysis: a case report and review of the literature

**DOI:** 10.1186/s40560-016-0193-9

**Published:** 2016-12-01

**Authors:** Toshihiko Yoshizawa, Kazuhiko Omori, Ikuto Takeuchi, Yuto Miyoshi, Hiroshi Kido, Etsuhisa Takahashi, Kei Jitsuiki, Kouhei Ishikawa, Hiromichi Ohsaka, Manabu Sugita, Youichi Yanagawa

**Affiliations:** 1 Department of Acute Critical Care Medicine, Shizuoka Hospital, Juntendo University, Tokyo, Japan; 2 1129 Nagaoka, Izunokuni City, Shizuoka 410-2295 Japan; 3 Tokushima University, Tokyo, Japan; 4 Juntendo University, Tokyo, Japan

**Keywords:** Heat stroke, Rhabdomyolysis, Rehabilitation

## Abstract

**Background:**

Severe heat stroke tends to be complicated with rhabdomyolysis, especially in patients with exertional heat stroke. Rhabdomyolysis usually occurs in the acute phase of heat stroke. We herein report a case of heat stroke in a patient who experienced bimodal rhabdomyolysis in the acute and recovery phases.

**Case presentation:**

A 34-year-old male patient was found lying unconscious on the road after participating in a half marathon in the spring. It was a sunny day with a maximum temperature of 24.2 °C. His medical and family history was unremarkable. Upon arrival, his Glasgow Coma Scale score was 10. However, the patient’s marked restlessness and confusion returned. A sedative was administered and tracheal intubation was performed. On the second day of hospitalization, a blood analysis was compatible with a diagnosis of acute hepatic failure; thus, he received fresh frozen plasma and a platelet transfusion was performed, following plasma exchange and continuous hemodiafiltration. The patient’s creatinine phosphokinesis (CPK) level increased to 8832 IU/L on the fifth day of hospitalization and then showed a tendency to transiently decrease. The patient was extubated on the eighth day of hospitalization after the improvement of his laboratory data. From the ninth day of hospitalization, gradual rehabilitation was initiated. However, he felt pain in both legs and his CPK level increased again. Despite the cessation of all drugs and rehabilitation, his CPK level increased to 105,945 IU/L on the 15th day of hospitalization. Fortunately, his CPK level decreased with a fluid infusion. The patient’s rehabilitation was restarted after his CPK level fell to <10,000 IU/L. On the 31st day of hospitalization, his CK level decreased to 623 IU/L and he was discharged on foot. Later, a genetic analysis revealed that he had a thermolabile genetic phenotype of carnitine palmitoyltransferase II (CPT II).

**Conclusions:**

Physicians should pay special attention to the stress of rehabilitation exercises, which may cause collapsed muscles that are injured by severe heat stroke to repeatedly flare up.

## Background

Severe heat stroke tends to be complicated with rhabdomyolysis, especially in patients with exertional heat stroke [1–4]. Rhabdomyolysis may lead to systemic effects, including the local occurrence of compartment syndrome, hyperkalemic cardiac arrest, and/or lethal disseminated intravascular coagulopathy [5–7]. Rhabdomyolysis usually occurs in the acute phase of heat stroke. We herein report a case of heat stroke in a patient who experienced bimodal rhabdomyolysis in the acute and recovery phases.

## Case presentation

**Fig. 1:**
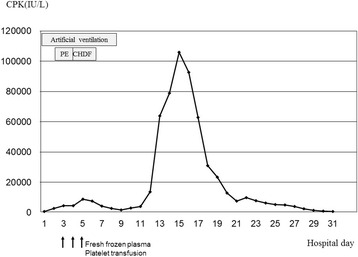
The time course of the changes in the patient’s creatinine phosphokinesis (CPK) data. The patient’s CPK level increased to 8832 IU/L on the fifth day of hospitalization and then showed a transient tendency to decrease. From the ninth day of hospitalization and following the start of rehabilitation, the patient’s CPK level increased again to reach 105,945 IU/L on the 15th day of hospitalization. *PE* plasma exchange, *CHDF* continuous hemodiafiltration

A 34-year-old male patient was found lying unconscious with a head injury on the road after participating in a half marathon in the spring. It was a sunny day with a maximum temperature of 24.2 °C and a humidity of 54%. A physician who was transported by helicopter to check on the patient reported that his Glasgow Coma Scale score was 6 and that he presented marked restlessness. His blood pressure was 110/80 mmHg, his heart rate was 140 beats per minute (BPM), his respiratory rate was 40 breaths per minute (BPM), and his axillary temperature was 40.8 °C. He was transported to our hospital by a ground ambulance after the infusion of a sedative agent and the rapid infusion of cooled lactated Ringer. His medical and family history was unremarkable. He did not have sign of flu in a few days. Upon arrival, his Glasgow Coma Scale score was 10. His blood pressure was 116/86 mmHg, his heart rate was 164 BPM, his respiratory rate was 36 BPM, his SpO_2_ level was 95% with oxygen (8 l/min by mask), and his bladder temperature was 40.2 °C. The physiological findings included hyperhidrosis with restless confusion. After the rapid infusion of 3500 ml of cooled lactated Ringer and gastric lavage with iced water, his bladder temperature decreased to 38.8 °C within 30 min of his arrival and the patient became calm. A chest roentgen revealed no abnormal findings, while an electrocardiogram showed sinus tachycardia without a change in the ST segments. Head CT, which was performed to determine the cause of the patient’s unconsciousness, revealed no brain abnormalities; however, the patient’s marked restlessness and confusion returned. To secure the patient’s safety, a sedative was administered and tracheal intubation was performed. The main results of a blood analysis are shown in Table 1. On the second day of hospitalization, a blood analysis revealed the following findings: aspartate aminotransferase (AST), 144 IU/L; alanine aminotransferase (ALT), 86 IU/L; prothrombin activation ratio, 22%; platelet count, 5 × 10^4^/mm^3^; and ammonia level, 108 μg/dl. These values were compatible with a diagnosis of acute hepatic failure (according to the Japanese guidelines) [8]; thus, he received fresh frozen plasma and a platelet transfusion was performed. On the third day of hospitalization, a blood analysis revealed the following findings: AST level, 14,894 IU/L; ALT level, 14,355 IU/L, prothrombin activation ratio, 43.8%; and platelet count, 3.8 × 10^4^/mm^3^; thus, plasma exchange was performed for 2 days, followed by continuous hemodiafiltration for 3 days. The time course of the changes in the patient’s creatinine phosphokinesis (CPK) levels is shown in Fig. 1. The patient’s CPK level increased to 8832 IU/L on the fifth day of hospitalization and then showed a tendency to transiently decrease. The patient was extubated on the eighth day of hospitalization, after showing the ability to respond to commands and the improvement of his laboratory data. From the ninth day of hospitalization, gradual rehabilitation was initiated; this included transferring to a wheelchair or standing at his bedside. However, he felt pain in both legs and his CPK level increased again. Despite the cessation of all drugs and rehabilitation, his CPK level increased to 105,945 IU/L on the 15th day of hospitalization. During this period, he had a low-grade fever ranging from 37.2 to 37.8 °C. Fortunately, his CPK level decreased with a fluid infusion, which was administered to prevent renal failure. The patient’s rehabilitation was restarted after his CPK level fell to <10,000 IU/L. On the 31st day of hospitalization, his CPK level decreased to 623 IU/L and he was discharged on foot. Later, a genetic analysis revealed that he had a thermolabile genetic phenotype of carnitine palmitoyltransferase II (CPT II).

**Table 1:** The laboratory analysis results

Arterial blood gas					
pH	7.374		pCO_2_	23.7 mmHg	
pO_2_	152 mmHg		Bicarbonate	13.5 mmol/l	
Cell blood count					
White blood count		8600/μl	Hematocrit	48.3%	
Platelet		19.2 × 10^4^/μl			
Serum biochemical data					
Aspartate aminotransferase		46 IU/l	Alanine aminotransferase		35 U/l
Lactate dehydrogenase		285 IU/l	Total bilirubin		0.6 mg/dl
Blood urea nitrogen		27.3 mg/dl	Glucose		101 mg/dl
Creatinine		2.38 mg/dl	Creatine phosphokinase		301 IU/l
Sodium		146 mEq/l	Chloride		113 mEq/l
Potassium		4.3 mEq/l	C reactive protein		0.3 mg/dl
Coagulation					
Activated partial thromboplastin time 22.1 s			Prothrombin time %		74%
Fibrinogen degradation products		9.3 μg/mL

### Discussion

We herein report a case of heat stroke in a patient with bimodal rhabdomyolysis in the acute and recovery phases. We performed a PubMed search to identify any related articles using the key words “heat stroke” and “rhabdomyolysis”. As a result, we found 110 articles about heat stroke with rhabdomyolysis. Among these cases, we found 17 cases involving individuals with heat stroke complicated with rhabdomyolysis in which the time course of the CPK level was described [1, 9–24]. We summarized these cases, including the present case, in Table 2. Among them, only two reports from Japan showed bimodal rhabdomyolysis [15, 22]. In one of these two reports, Takahashi et al. described a 16-year-old male patient who experienced convulsions 3 days after living donor liver transplantation [22]. After the convulsions on postoperative day 5, the patient’s CPK level, which had been showing a tendency to decrease, increased from 715 to 24,985 IU/L. Convulsions can cause rhabdomyolysis; thus, this case report was excluded from the studies that described the natural course of bimodal rhabdomyolysis induced by heat stroke [25]. Two reports by Miura et al. described the case of 38-year-old man who experienced a life-threatening flare-up of rhabdomyolysis (CPK level of 84,612 IU/L on the third hospital day) and who was treated by plasma exchange, hemodiafiltration, steroid pulse therapy, and anticoagulant treatment [15]. His general condition was initially thought to be improving; however, his smoldering rhabdomyolysis suddenly flared up with a marked increase in his CPK level (105,231 IU/L on the 18th day of hospitalization) when the steroid dosage was reduced and rehabilitation was initiated. Thereafter, his condition rapidly deteriorated and he eventually died, despite the provision of aggressive treatment. In addition, Fink et al. reported the case of a 16-year-old male athlete with heat stroke and rhabdomyolysis [19]. The patient survived and was discharged on day 14, but his CPK level was more than 1000 IU/L for several weeks after his discharge. Their report did not indicate whether the patient’s rhabdomyolysis was bimodal. Similarly to our case, in the four Japanese reports of six patients who suffered bimodal rhabdomyolysis in the acute and recovery phases (more than 2 weeks after severe heat stroke), all of the patients could survive and start rehabilitation (Table 3) [26–29]. Accordingly, the authors’ hypothesized that during the recovery phase, the stress of rehabilitation exercises can cause collapsed muscles that are injured by heat stroke to repeatedly flared up. Drugs that are administered during intensive treatment in the acute phase may be involved in the occurrence of bimodal rhabdomyolysis. However, this possibility was considered to be unlikely in the present case because drug-induced rhabdomyolysis usually subsides when the drugs are stopped [30]. In our search of the literature, heat stroke-induced bimodal rhabdomyolysis was only described in Japanese case reports; thus, genetic differences may affect this phenomenon.

**Table 2:** A summary of the reports on heat stroke in which the time course of rhabdomyolysis was described

No	Reporter	Year	Age	Sex	Trigger	1st peak D	CPK max	HD	Outcome	Bimodal	2nd peak D	CPK max	Trigger
1	Wu	2015	27	Male	Exercise	2	55,650	Yes	Survival	No			
2	Asserraji	2014	35	Male	Marathon	5	91,596	Yes	Death	No			
3	Raj	2013	11	Male	Jog	1	4326	Yes	Survival	No			
4	Horseman	2013	22	Male	Walking	1	649,530	Yes	Survival	No			
5	Azzopardi	2012	25	Male	Marathon	2	178,850	No	Survival	No			
6	Muñiz	2012	15	Male	Football	2	39,954	No	Survival	No			
7	Trujillo	2011	14	Female	Exercise	3	36,423	Yes	Survival	No			
8	Lin	2011	11	Female	Jogging	2	21,351	No	Survival	No			
9	Miura	2010	38	Male	Marathon	3	84,612	No	Death	Yes	18	105,231	Reha
10	Lee	2010	57	Male	Kot spring	3	9565	Yes	Death	No			
11	Niu	2009	47	Male	Labor	1	4682	No	Survival	No			
12	Akieda	2008	75	Male	Bath	3	4299	No	Survival	No			
13	Fink	2006	16	Male	Football	3	90,720	No	Survival	?	>2 wks	>1000	Discharge
14	Broessner	2005	38	Male	Hiking	4	1024	No	Survival	No			
15	Wakino	2005	23	Male	Labor	5	620,920	Yes	Survival	No			
16	Takahashi	2005	16	Male	Rugby	?	?	Yes	Survival	Yes	8	24,985	Convulsion
17	Pechlaner	2002	28	Male	Labor	2	1920	No	Survival	No			
18	Present		28	Male	Marathon	5	8832	Yes	Survival	Yes	22	9230	Reha

? means not described, *D* day, *Reha* rehabilitation, *wks* weeks, *CPK* creatinine phosphokinesis, *HD* hemodialysis, *max* maximum

**Table 3:** The Japanese reports of bimodal rhabdomyolysis after heat stroke

No	Reporter	Year	Age	Sex	Trigger	1st peak D	CPK max	HD	Outcome	Bimodal	2nd peak D	CPK max	Trigger
1	Suzuki	1996	23	Male	Training	3	300,762	Yes	Survival	Yes	20	14,154	Reha
2	Kajiwara	1993	17	Male	Jogging	3	>15,000	No	Survival	Yes	22	4500	Reha
3	Kuriyama	1990	19	Male	Jogging	6	10,425	No	Survival	Yes	15	105,945	Reha
4	Nagao	1985	21	Male	Jogging	6	5570	No	Survival	Yes	18	474	Reha
5	Nagao	1985	18	Male	Jogging	5	4530	No	Survival	Yes	19	9800	Reha
6	Nagao	1985	16	Male	Kendo	3	9410	No	Death	Yes	17	309,000	Reha

*D* day, *CPK* creatinine phosphokinesis, *HD* hemodialysis, *max* maximum

CPT II is a pivotal enzyme in mitochondrial fatty acid oxidation, which is essential for energy production during simultaneous glucose sparing and a requirement for major energy supply, such as during prolonged fasting or exercise [31]. Cases with the thermolabile genetic phenotype of CPT II have been described mainly in Japan and China. Recent studies have suggested the association of this phenotype with influenza-associated encephalopathy, encephalopathy during a high-grade fever caused by human herpesvirus-6, enterovirus 71, Echo virus, Coxsackievirus, rotavirus, respiratory syncytial virus, adenovirus infection, or sudden unexpected death in infancy [31–40]. Generally, CPT II deficiency has three clinical presentations: a lethal neonatal form, a severe infantile hepatocardiomuscular form, and a myopathic form (which is usually mild and can manifest from infancy to adulthood) [41]. While the former two are severe multisystemic diseases characterized by liver failure with hypoketotic hypoglycemia, cardiomyopathy, seizures, and early death, the latter is characterized by recurrent exercise-induced muscle pain and weakness, sometimes associated with myoglobinuria, resembling our case [41]. The myopathic form of CPT II deficiency is the most common disorder of lipid metabolism affecting the skeletal muscle and is the most frequent cause of hereditary myoglobinuria, and males are more likely to be affected than females [41]. Accordingly, the thermolabile genetic phenotype of CPT II in the present case might have affected the occurrence of bimodal rhabdomyolysis, even during mild exercise like rehabilitation after depletion of energy in the muscle due to an initial attack of heat stroke [42]. Oda et al. also suggested that the thermolabile genetic phenotype of CPT II was a risk factor for severe heat stroke [43]. Like Reye syndrome, heat stoke induced by thermolabile genetic phenotype of CPT II might be classified as a fatty acid oxidation disorder in the future [44].

## Conclusions

Physicians should pay special attention to the stress of rehabilitation exercises, which may cause collapsed muscles that are injured by severe heat stroke to repeatedly flare up.

## Abbreviations

AST: Aspartate aminotransferase
CHDF: Continuous hemodiafiltration
CPK: Creatinine phosphokinesis
PE: Plasma exchange
